# The Application of a Macroinvertebrate Indicator in Afrotropical Regions for Pesticide Pollution

**DOI:** 10.1155/2018/2581930

**Published:** 2018-09-12

**Authors:** Wynand Malherbe, Johan H. J. van Vuren, Victor Wepener

**Affiliations:** ^1^Water Research Group, Unit for Environmental Sciences and Management, North-West University, Private Bag X6001, Potchefstroom 2520, South Africa; ^2^Department of Zoology, University of Johannesburg, P.O. Box 524, Auckland Park 2006, South Africa

## Abstract

Many biotic integrity indices are not able to isolate community effects due to pesticide exposure as the communities also respond to other anthropogenic and natural stressors. A macroinvertebrate trait bioindicator system that is pesticide specific was therefore developed to overcome these challenges. This system, called SPEAR (SPEcies At Risk), was applied in South Africa as an indicator to link known pesticide catchment usage to changes in the macroinvertebrate community, especially when analytical methods are inconclusive. In addition, the SPEAR_salinity_ index within the SPEAR suite of tools was also evaluated for its effectiveness in South Africa. The results indicated that all of the sites have either been exposed to the same pesticide pressure or not been exposed to pesticides as the SPEAR results were similar when compared to the pesticide intensity. The interaction with other factors like nutrients or salinity was likely a factor that confounded the SPEAR_pesticides_ indicator.

## 1. Introduction

Chemical monitoring of aquatic ecosystems is often insufficient to determine quality as it does not take into account higher level effects on biota, in-stream speciation of chemicals, interactions with other physical impacts, and variations due to longitude and time [1]. To overcome this, in-stream biota are used as environmental indicators as they can integrate all of these higher level effects [2]. In South Africa, the SASS5 macroinvertebrate index [3] has been used extensively in the monitoring of water quality within streams [4, 5]. This index makes use of family level macroinvertebrate data, and sensitivity scores for each taxon have been calculated based on the derived sensitivity to water quality changes [3]. However, the index is not able to differentiate macroinvertebrate community effects due to specific stressors as the communities often respond to a range of anthropogenic as well as natural stressors [6].

Therefore, the use of macroinvertebrate traits has been proposed as indicators of community effects due to specific stressors rather than applying a purely taxonomic approach [7]. Studies on organic contaminants [8, 9], pesticides [10], salinity [11], and more recently metals [12, 13] have shown the value of using biological traits in ecosystem assessment. One of these approaches is the SPEcies At Risk (SPEAR) system that was developed as a bioindicator system making use of macroinvertebrate traits that are pesticide specific to link pesticide exposure to macroinvertebrate community responses [10, 14].

Preliminary pesticide risk assessments in two intensive irrigation systems in South Africa were conducted using the predictive software models PRIMET and PERPEST [15, 16]. These studies identified numerous pesticides (between 15 and 20 active ingredients) (e.g., pyrethroids like deltamethrin and carbamates, as well as various insecticides and pesticides) that presented a moderate to high risk to aquatic macroinvertebrates in these systems together with the predicted effect concentrations based on the pesticide properties, application scheme, and aquatic ecosystem specifications. PERPEST results for deltamethrin indicated that a high probability of effects is expected on insects and micro- and macrocrustacean communities [16]. Exposure validation was conducted through targeted pesticide residue analysis in the irrigation systems of Vaalharts [16] and Crocodile River (West) [15]. However, in both instances the pesticide levels were below detection limits (0.02 *μ*g/g) and therefore pesticide exposure could not be directly linked to biological responses. Therefore, the aim of this study was to determine whether the SPEAR index system would be applicable within an Afrotropical region. The applicability was determined by testing the following hypotheses: Eurasia and Australian SPEAR databases will provide similar results; SPEAR_pesticides_ will show a positive correlation with the increased pesticide intensity in study area; and the SPEAR_pesticides_ will not be correlated with EC and a local biotic index.

## 2. Materials and Methods

Macroinvertebrate sampling was undertaken at sites associated with two large agricultural irrigation schemes in South Africa. The Vaalharts Irrigation Scheme is situated in the semiarid region while the Crocodile River (West) Irrigation Scheme was situated in a subtropical region. Sampling was conducted at preselected sites above (upstream), adjacent to, and below (downstream) the irrigation system between 2005 and 2009. Seasonal variability and influence of pesticide run-off were studied by sampling during the rainy (summer) and dry (winter) seasons. Sampling methodology as set out for the SASS5 biotic index [3] was applied using a 0.5 mm mesh sweep net and sampling marginal vegetation, substratum, and stones in and out of the current. Macroinvertebrates were fixed with 10% buffered formalin containing the vital stain, Rose Bengal. The samples were cleaned, identified to family level, and enumerated.

The family diversity was used to calculate SASS5 index values, based on the sensitivity rating scores [3] and SPEAR index scores. Both the SPEAR_pesticides_ index, designed for agricultural pesticides occurring in water in short-term pulses (Liess et al. 2005), and the SPEAR_salinity_ index, indicating continuous exposure to salinity [11], were calculated. The SPEAR calculator (www.systemecology.eu/SPEAR/) was used to determine the SPEAR_pesticides_ index. For the purposes of this study, it was decided to use both the European and Australian SPEAR_pesticides_ databases to determine if there are any differences between the results. This would in turn help to determine which database would be better suited for application within South Africa. Beketov et al. [17] provided categories for the ecological status of the macroinvertebrates in Europe based on the SPEAR index score. The categories range from high (SPEAR > 44) to good (SPEAR = 33 – 44), moderate (SPEAR = 22 – 33), poor (SPEAR 11 -22), and bad (SPEAR = < 11). In addition,* in situ* physicochemical parameters (electrical conductivity (EC), pH, temperature, oxygen concentration, and total dissolved solids (TDS)) were measured using a WTW water quality meter.

The correlations between EC and the SPEAR system indices were tested using linear regression and correlation analyses (Pearson correlation analysis using SPSS) similar to the methods applied by Schäfer et al. [11]. In addition, the relationship between the SPEAR index values and the SASS5 biotic index scores as well as an ordinal pesticide intensity measure (site position in relation to increased pesticide intensity) was tested. The significance of these relationships were tested at p < 0.05.

## 3. Results and Discussion

**Figure 1:**
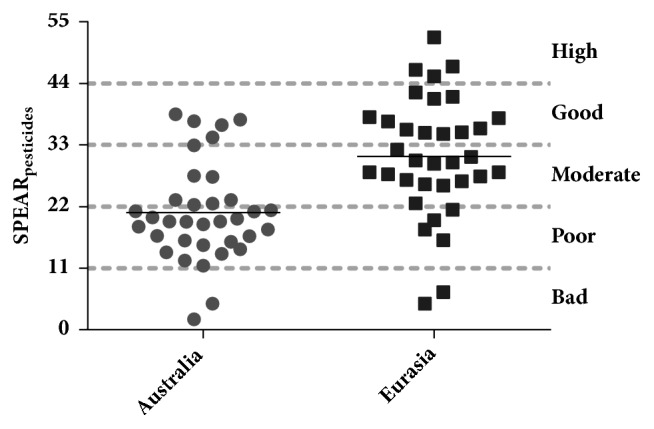
Comparison of the SPEAR_pesticides_ results for the selected sites using the European and Australian databases. The classification categories of Beketov et al. [17] are indicated by dashed lines.

The SPEAR system has been applied successfully in various parts of Europe and Asia as well as in Australia. The availability of trait databases is abundant in North America and Europe but data from the southern hemisphere are limited [11]. These authors adapted the European database with information available from Australia. Since South Africa has a similar climate range to Australia, the data collected during this study were subjected to both the European and Australian trait databases. Although the Australian trait database is based on temperate and Mediterranean climate, the Eurasia database was also based on different climatic conditions in Europe, with some climates similar to what was found in this study area. Ideally, a South African specific database would be needed to account for the specific climatic conditions throughout the study area and South Africa. More recently, the index was also applied in the mild climatic conditions of the Argentinean Pampas where it performed well in identified pesticide effects on stream macroinvertebrates [18]. The SPEAR_pesticide_ results indicated significant differences (p < 0.001) when using the European and Australian macroinvertebrate database (Figure 1) with the majority of sites occurring in the moderate to good category when using the Eurasian database. In contrast, when using the same classification scale for the Australian results, the majority of sites fell in the poor category.

**Figure 2:**
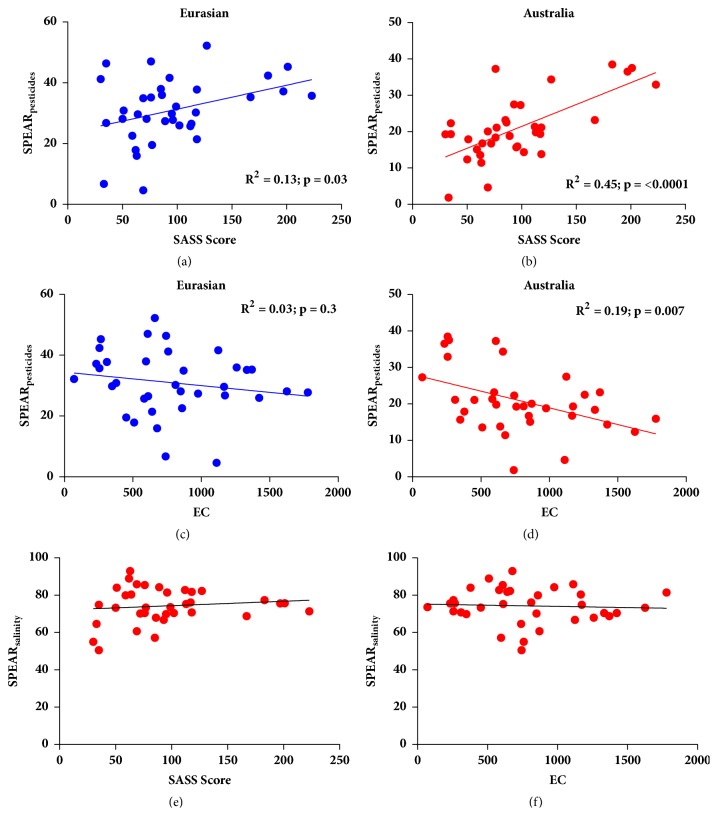
Linear regression indicating the relationships for (a) SPEAR_pesticides_ (Eurasian database) and (b) SPEAR_pesticides_ (Australian database) versus a biotic index, SASS5; (c) SPEAR_pesticides_ (Australian database) and (d) SPEAR_pesticides_ (Eurasian database) with the electrical conductivity (EC); (e) SPEAR_salinity_ versus the SASS5 scores; and (f) SPEAR_salinity_ versus EC.

The linear regression and Pearson correlation analysis of the SASS5 biotic index with the SPEAR_pesticide_ indicator (Figures 2(a) and 2(b)) indicated a significant correlation for both Australian and Eurasian databases. National river monitoring using the SASS5 biotic index has indicated that macroinvertebrate communities in this river range from moderately to largely modified [16]. Thus, these results indicate that there were differences in the databases and as such this hypothesis is rejected. In addition, an ordinal scale was used to represent predicted pesticide intensity with 1 being the least intense pesticide use to 5 being the most intense use of pesticide. It was assumed that pesticide intensity would increase downstream of the irrigation schemes that are known to use significant volumes of pesticides [19]. A linear regression indicated a negative correlation to this intensity measure and the SPEAR_pesticide_ indicator (R = 0.264) but this relationship was not significant. Thus, no clear evidence was found to indicate that SPEAR_pesticide_ differences are due to pesticide pollution and not general river decline.

Salinity, specifically increasing salinity, has been identified as an environmental problem in South Africa; it has the potential to influence pesticide effects while it is also known for increasing downstream of agricultural return flows [20, 21].* In situ* water quality measurements indicated that EC, TDS, and pH generally increased downstream of the irrigation schemes while oxygen and temperature parameters were similar at all sites. Therefore, the SPEAR_pesticide_ indicators were also correlated with the EC at the various sites to determine if there was any interaction with salinity. The regression analysis of the EC with the SPEAR_pesticide_ indicator (Figures 2(c) and 2(d)) showed no significant relationship for the results using the Eurasian database but the results using the Australian database did show significant correlation with the EC. Previously, when using the Australian database, no interactive effect between EC and SPEAR was found in a temperate setting in Australia [22]. Regression analysis for the SPEAR_salinity_ indicator (Figures 2(e) and 2(f)) based on the Australian database is presented to determine if the indicator is viable to detect changes in macroinvertebrate community structure due to salinity. No relationships were present when comparing the SPEAR_salinity_ to the EC (Figure 2(e)) as well as the SASS5 results (Figure 2(f)).

The spatial scales in this study in South Africa were relatively small compared to the European studies even though the sites were located in different climate and ecoregions. The biological traits used in the system are traits that will be responsive to pesticide effects. Numerous validation studies (in various regions in Europe) of the SPEAR system have shown that it is sensitive to pesticide contamination, relatively independent of abiotic factors, and applicable across different biogeographical regions in Europe [8–10, 23, 24] as well as in Australia [11] and Argentina [18]. In most of these studies it was possible to distinguish between the effects of pesticides and other stressors as well as the natural variation over large spatial scales [11]. However, in our study (even though it was a small sample size) it was difficult to distinguish between the impacts of pesticides and other confounding factors like habitat modification and other water quality changes, i.e., eutrophication and salinization (as measured with the SASS5 biotic index).

One of the criteria that the SPEAR system is based on, is the sensitivity score of the organisms to pesticides, specifically insecticides. However, many insecticides (and other pesticides) have different modes of action which influence the sensitivity of organisms to insecticides. This raises questions in terms of the effectivity of SPEAR [25]. Firstly, the sensitivity scores are not adequate to reflect the taxa found in Afrotropical or semi-arid subtropical systems. Differences in this study between Australian and Eurasian databases can possibly be attributed to the differing sensitivities between the two continents. However, recent work has indicated that differences in sensitivity could be limited depending on the taxa and the chemicals that are used [26, 27]. Secondly, is the system sensitive enough to detect pesticide specific signals rather than general stress within the macroinvertebrate community within the ecosystem? The potential stressors that are dominant at the selected sites in this study include salinity, nutrients, and habitat alteration [28]. The correlation with SASS5 results and the pesticide intensity measure indicated that the SPEAR_pesticide_ indicator could be useful to indicate the effects of pesticides on the aquatic community even though the relationships in this study was not significant. The negative correlation of EC with the SPEAR_pesticides_ indicated that salinity could be interacting with pesticide effects. Some studies have looked at the interaction of salinity with pesticides by using the SPEAR system [22]. That study in Australia did not find any evidence of interaction between salinity and pesticides, but it did indicate that salinity and pesticides were a major factor affecting the macroinvertebrate community structure.

Thirdly, the SPEAR system generally only looks at the acute effects of the insecticides and not chronic effects necessarily. Recently, Rico and van den Brink [25] developed an approach to assess relative sensitivity of macroinvertebrates to five different insecticide classes (organophosphates, pyrethroids, carbamates, organochlorines, and neonicotinoids) (Rubach et al. 2010) with four different modes of action. The approach also included the relationship of the relative sensitivity to selected biological traits that are important for short-term sensitivity to pesticides. In addition, the study looked at biological traits that are responsible for recovery of macroinvertebrate populations. These metrics were used to devise a ranking system that identifies vulnerable taxa that should then be included in higher tier risk assessments [25],

To evaluate the salinity effect on macroinvertebrate community structure in a similar approach to pesticides, Schäfer et al. [11] also compiled a trait database for salinity which resulted in the SPEAR_salinity_ system. The SPEAR_salinity_ system indicated a reasonably high relationship with logarithmic electrical conductivity (EC) as a salinity measurement for field monitoring data from Victoria and South Australia [11]. Other biotic indices used did not show any significant relationships with the logarithmic EC. Furthermore, the SPEAR_salinity_ index did not show any significant response to other water quality parameters indicating its selectivity towards salinity [11]. Van den Brink et al. [7] also indicated that the SPEAR_salinity_ indicator is a promising tool to use as an indicator of community or trait responses of macroinvertebrate river communities to salinity as a driver of change. In future, the SPEAR_salinity_ approach should be tested more extensively for its effectiveness in South Africa as many systems here are already either experiencing salinization or at risk of future salinization. However, as with the SPEAR_pesticide_ system, the trait database for salinity will be an important consideration when applying the system.

The effects of pesticides on macroinvertebrates in South Africa have been poorly studied in the past [29] and therefore the database used to derive the SPEAR index scores should be improved. In many cases the analyses of pesticides are inconclusive due to high levels of detection, confounding factors, cost, and human resources. The SPEAR system indicated promise to identify pesticide exposure using the macroinvertebrate community as it overcomes many of the previously mentioned factors. However, more research and fine tuning of specifically the trait database are needed to be used successfully. Currently, the information available on invertebrate traits specifically related to pesticides is extremely limited; although more information is available in terms of invertebrate traits and salinity [30] and as such research into the applicability of SPEAR_salinity_ should also be increased in future. The improvement in trait databases should focus on publishing available databases, including different traits, increasing taxa, and life stages as well as the stressor response to the various traits. In addition to the improvement in trait databases, van den Brink et al. [31] indicated that to improve the usefulness of trait-based methods these traits should be analysed to determine their response to various physical and chemical stressors (on their own as well as in combination). Comparisons should also be made between existing risk assessments that made use of ecotoxicology and bioassessment approaches [15, 16] so that the limitations and advantages of trait-based assessment can be further refined.

## Data Availability

All of the data used within this manuscript were generated within this study. The SPEAR databases for Europe and Asia were accessed online at www.systemecology.eu/SPEAR/.
